# Band-driven switching of magnetism in a van der Waals magnetic semimetal

**DOI:** 10.1126/sciadv.adk1415

**Published:** 2024-04-12

**Authors:** Hideki Matsuoka, Shun Kajihara, Takuya Nomoto, Yue Wang, Motoaki Hirayama, Ryotaro Arita, Yoshihiro Iwasa, Masaki Nakano

**Affiliations:** ^1^ RIKEN Center for Emergent Matter Science (CEMS), Wako 351-0198, Japan.; ^2^ Quantum-Phase Electronics Center and Department of Applied Physics, The University of Tokyo, Tokyo 113-8656, Japan.; ^3^ Research Center for Advanced Science and Technology, The University of Tokyo, Tokyo 153-8904, Japan.

## Abstract

Magnetic semimetals form an attractive class of materials because of the nontrivial contributions of itinerant electrons to magnetism. Because of their relatively low–carrier-density nature, a doping level of those materials could be largely tuned by a gating technique. Here, we demonstrate gate-tunable ferromagnetism in an emergent van der Waals magnetic semimetal Cr_3_Te_4_ based on an ion-gating technique. Upon doping electrons into the system, the Curie temperature (*T*_C_) sharply increases, approaching near to room temperature, and then decreases to some extent. This non-monotonous variation of *T*_C_ accompanies the switching of the magnetic anisotropy, synchronously followed by the sign changes of the ordinary and anomalous Hall effects. Those results clearly elucidate that the magnetism in Cr_3_Te_4_ should be governed by its semimetallic band nature.

## Teaser

Non-monotonous carrier-density dependence of ferromagnetism associated with a semimetallic-band nature is demonstrated.

## INTRODUCTION

Research on itinerant magnetism has a long history starting from the ancient period, but it continues to be a major topic in modern condensed matter physics. Of particular importance is to understand an interplay between itinerant electrons and local magnetic moments, which provides an insight into the origin of magnetism. As for a material system, magnetic semimetals form an important branch, where the intersection of electronic bands with different spin and orbital characters plays an essential role for their nontrivial magneto-transport properties. The unique features of this system have been demonstrated in magnetic topological insulators (*1*–*4*), magnetic Weyl semimetals (*5*–*7*), ferromagnetic/antiferromagnetic Kagome systems (*7*–*9*), and magnetic two-dimensional (2D) materials (*10*–*13*), where an interplay between the crossing points of the electronic bands and local magnetic moments leads to the unprecedented magneto-transport phenomena as represented by the (half-)quantization of the anomalous Hall (AH) effect (AHE) or giant AH angle, which is associated with the emergence of the Berry curvature at the band-crossing points (*14*–*17*). On the other hand, in those systems, the origin of magnetism has been of great interest as well. In particular, the unique carrier-density dependence of the Curie temperature (*T*_C_) in magnetic topological insulators has been attributed to the contributions of itinerant Dirac electrons to magnetism (*4*, *18*), which should be relevant to the energetic stability of the spontaneous magnetic ordering. These unique features could be generally observed in magnetic semimetals hosting both electron- and hole-like bands that are crossing near the Fermi level (*E*_F_), but so far, such a discussion has been mostly limited to magnetic topological systems. To deepen our knowledge on the origin of magnetism in magnetic semimetals, it is highly desired to explore other material systems and examine the impact of a semimetallic band nature on the overall magnetic properties.

In this study, we focus on Cr_3_Te_4_ as an ideal material platform. As will be introduced later, Cr_3_Te_4_ is an emergent van der Waals (vdW) magnetic semimetal, whose magnetic properties are expected to be strongly coupled to its semimetallic band nature. To systematically control a doping level (i.e., *E*_F_) in Cr_3_Te_4_, we apply an electrical gating technique. The gate-controlled tuning of magnetism has been rather established approach, providing notable achievements both in magnetic semiconductors and even in magnetic metals (*19*–*23*), where *T*_C_ could be largely enhanced by increasing the carrier density. However, in the case of magnetic semimetals, we could expect to have nontrivial contributions of itinerant electrons near the band-crossing points, which might provide an anomalous carrier-density dependence of their magnetism.

## RESULTS

### Basic properties of Cr_3_Te_4_ epitaxial thin films

**Fig. 1.**
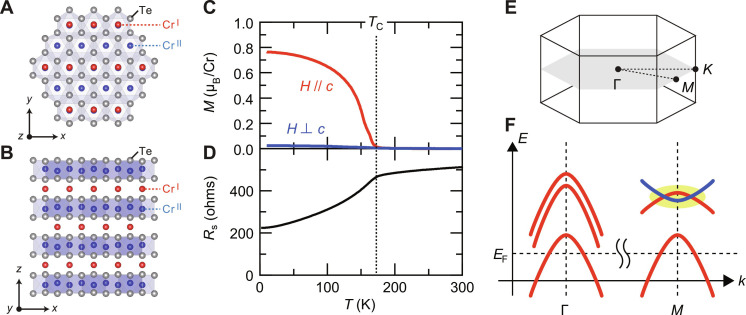
Basic properties of Cr_3_Te_4_ epitaxial thin films. (**A** and **B**) Schematic (A) top and (B) side views of the crystal structure of Cr_3_Te_4_ drawn by VESTA (*51*). (**C**) The magnetization versus temperature (*M-T*) curves of a typical Cr_3_Te_4_ epitaxial film taken in the field-cooling conditions with the out-of-plane (*H // c*, red) and in-plane (*H* ⊥ *c*, blue) magnetic fields. The magnitude of the applied field was μ_0_*H* = 10 Oe. The layer number of this particular sample was 50, which is defined as the number of the host CrTe_2_ layer. (**D**) The corresponding sheet resistance versus temperature (*R*_s_*-T*) curve of the same sample taken at zero field. The vertical dashed line corresponds to the Curie temperature (*T*_C_), which is defined as the onset temperature of the spontaneous magnetization. Note that *T*_C_ could be well-defined in the *R*_s_*-T* curve as a kink temperature, which is used to probe *T*_C_ in the gating experiments. (**E** and **F**) Schematic (E) first Brillouin zone and (F) band structure of Cr_1+δ_Te_2_ as revealed by the angle-resolved photoemission spectroscopy measurements (*35*), where electron- and hole-like bands intersect with each other to form a semimetallic band region near the Fermi level (*E*_F_) at the *M* point in the momentum space (yellow-colored area).

Cr_3_Te_4_ is one of the self-intercalated phases of chromium telluride (Cr_1+δ_Te_2_) (*24*), consisting of the host 2D CrTe_2_ layers and the intercalated Cr layers that form the 1 × 2 superstructure within the vdW gap as illustrated in Fig. 1 (A and B). Cr_1+δ_Te_2_ is a rather classical material system (*25*–*27*), but its relatively high *T*_C_ has attracted considerable interest in the context of “2D magnet” toward potential 2D spintronics applications, and a growing number of papers has been published very recently including demonstrations of room-temperature 2D ferromagnetism (*28*–*31*). On the other hand, recent fundamental studies on Cr_1+δ_Te_2_ epitaxial thin films grown by molecular-beam epitaxy (MBE) have demonstrated their intriguing magnetic properties, which are even distinct from those of their bulk counterparts (*32*–*35*). In this study, we started a series of the gating experiments with the metastable ferromagnetic phase of Cr_3_Te_4_ epitaxial thin films characterized with *T*_C_ of ~160 K and large out-of-plane magnetic anisotropy, which were prepared by MBE by following our previously established growth process (see Materials and Methods) (*34*).

Figure 1C shows the magnetization versus temperature (*M-T*) curves of a typical sample taken with the out-of-plane (red) and in-plane (blue) magnetic fields. The *M-T* curve with the out-of-plane field showed substantially larger signals than that with the in-plane field, indicating its large out-of-plane magnetic anisotropy, which is one of the unique features of the metastable ferromagnetic phase of Cr_3_Te_4_ epitaxial thin films (*34*). *T*_C_ of this particular sample was determined to be 170 K, corresponding to the onset temperature of the *M-T* curve. This onset of ferromagnetism could be well-defined in the sheet resistance versus temperature (*R*_s_*-T*) curve as a “kink” temperature (Fig. 1D), which enables us to track the variation of *T*_C_ with increasing a doping level in the following gating experiments. Figure 1 (E and F) illustrates a schematic band structure of Cr_1+δ_Te_2_ epitaxial thin films as revealed by the angle-resolved photoemission spectroscopy measurements (*35*), which verified the existence of a characteristic semimetallic band region near *E*_F_, where electron- and hole-like bands intersect with each other. The crossing points are accessible by a thermal-annealing treatment, which results in switching *T*_C_ from 160 K to above room temperature and magnetic anisotropy from the out-of-plane easy-axis type to the in-plane easy-plane type (*32*, *33*, *35*). Those results imply that the carrier density should play an important role for the magnetism in Cr_1+δ_Te_2_, although the effect of a thermal-annealing treatment remains unclear.

### Gate-tunable ferromagnetism in Cr_3_Te_4_

**Fig. 2.**
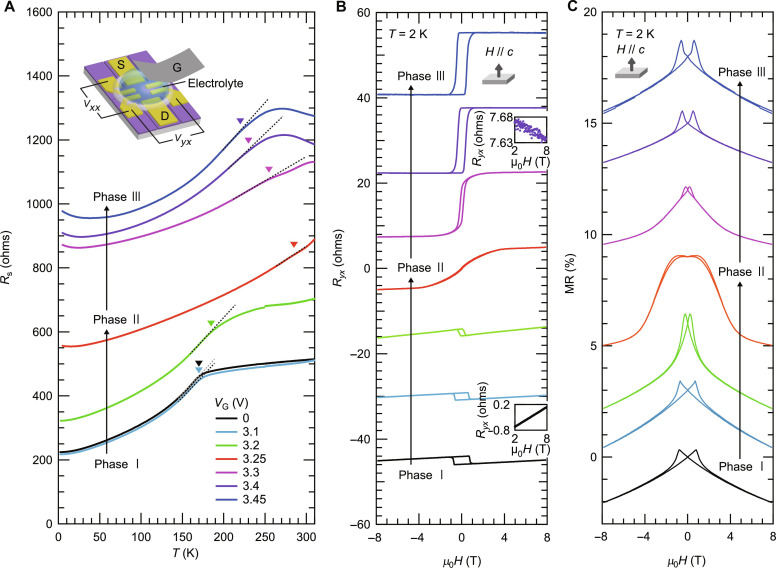
Gate-tunable ferromagnetism in Cr_3_Te_4_. (**A**) The *R*_s_*-T* curves of a typical device (device A) taken at different gate voltages (*V*_G_). The inset shows a schematic device structure with a top-gate configuration. Phase I, phase II, and phase III are defined as the *V*_G_ regions of 0 to ~3.1 V, 3.2 to ~3.3 V, and 3.4 to ~3.45 V, respectively. The inverse triangles correspond to *T*_C_, which is defined from the kink temperature in the *R*_s_-*T* curve. (**B** and **C**) The magnetic-field dependences of (B) the anti-symmetrized Hall resistance (*R_yx_*) and (C) the symmetrized magnetoresistance (MR) of device A taken at *T* = 2 K at each *V*_G_. The magnetic fields were set to be the out-of-plane directions. The inset two panels in (B) are the magnified views of *R_yx_* in the field range above 2 T for two representative voltages, *V*_G_ = 0 V and *V*_G_ = 3.4 V, demonstrating the sign change of the ordinary Hall effect (OHE) at a highly electron-doped regime. The MR is defined as [*R_xx_*(μ_0_*H*) − *R_xx_*(μ_0_*H* = 0 T)]/*R_xx_*(μ_0_*H* = 0 T), where *R_xx_* corresponds to the symmetrized longitudinal resistance. All the *R_yx_* and MR data are vertically shifted for clarity except for the data in the inset two panels in (B).

To tune a doping level largely across the semimetallic band region, we chose an in situ Li intercalation technique, which has been widely used for controlling physical properties of a variety of layered materials through a reversible carrier doping process (*22*, *36*–*38*). We fabricated ion-gating devices with a top-gate configuration (see the inset of Fig. 2A) by using a polymer electrolyte with LiClO_4_. The details of the device fabrication process and the gating procedure are described in Materials and Methods. Figure 2A shows the *R*_s_*-T* curves of a typical device taken at different gate voltages (*V*_G_), providing an overview of the gating effects in Cr_3_Te_4_. Above a threshold *V*_G_, *R*_s_ started to increase monotonously with increasing positive *V*_G_, which corresponds to electron doping. This behavior is quite reasonable considering that Cr_3_Te_4_ is a hole-type conductor (*34*), and the increase of *R*_s_ could be attributed to the depletion of hole carriers. We observed a notable modulation of the kink temperature above a threshold *V*_G_, which appeared to be non-monotonous against *V*_G_ and approaching near to room temperature at the intermediate regime (*V*_G_ = 3.25 V). In the following, we categorize the obtained results into three regions, phase I (*V*_G_, 0 to ~3.1V), phase II (*V*_G_, 3.2 to ~3.3 V), and phase III (*V*_G_, 3.4 to ~3.45 V) and discuss the magnetic properties at each phase based on the magneto-transport results. We note that the observed gating effects were highly reversible and reproducible among different devices (see Supplementary Text), suggesting that electron doping was induced by a reversible Li intercalation process rather than irreversible chemical reactions such as Te removal.

Figure 2B demonstrates the variation of the anti-symmetrized Hall resistance (*R_yx_*) versus magnetic field curves with increasing a doping level taken at the lowest temperature (*T* = 2 K) at each *V*_G_. The magnetic fields were set to be the out-of-plane direction. *R*_*yx*_ of a magnetic material is usually known to have two components, the ordinary Hall component proportional to the external magnetic fields and the AH component proportional to the magnetization. In Fig. 2B, *R_yx_* above 2 T was linear to the external fields for all *V*_G_, which could be attributed to the ordinary Hall effect (OHE). With increasing *V*_G_, OHE eventually showed a sign change from positive to negative as highlighted in the inset of Fig. 2B for two representative voltages, *V*_G_ = 0 V and *V*_G_ = 3.4 V, evidencing that the carrier type was changed from holes to electrons in Cr_3_Te_4_ upon electron doping. As will be explained later, the slope of OHE corresponding to the Hall coefficient (*R*_H_) exhibited non-monotonous behavior against *V*_G_ with a peak-like structure within phase II, and the sign change occurred at the boundary between phase II and phase III. In addition, we verified that the sign of AHE was also switched from negative to positive exactly at a specific *V*_G_ where *R*_H_ recorded the maximum value in phase II, suggesting their correspondence. These sign changes of OHE and AHE occurred continuously without introducing an insulating phase at the intermediate regime as shown in Fig. 2A. This directly proves that a semimetallic band region hosting both electron- and hole-like bands illustrated in Fig. 1F should exist in the present sample as well and that *E*_F_ should pass through this region within the applied *V*_G_ range. Moreover, the observed quite systematic and synchronous evolutions in OHE and AHE suggest that AHE should have the intrinsic origin associated with the band structure, and that there should be a singularity of the Berry curvature in the momentum space near *E*_F_, most likely associated with the crossing points in the semimetallic band region.

Now, we turn to focus on the shape of AHE. We found that the evolution of the shape of AHE occurred in two steps. At phase I, a square-shaped AHE with a sizable hysteresis loop was observed, corresponding to the out-of-plane magnetic anisotropy. On the other hand, this hysteresis completely disappeared at phase II and again opened up at phase III. The observed nonlinear behavior without hysteresis at phase II could be attributed to AHE with the in-plane magnetic anisotropy, where the AHE signal should increase gradually as the magnetization direction is changed from the in-plane direction at zero field to the out-of-plane direction at finite out-of-plane external fields. This series of changes in AHE across the three phases should therefore suggest that the magnetic anisotropy was changed from the out-of-plane type to the in-plane type and lastly switched back to the out-of-plane one. Such a sharp, complete, and consecutive switching of the magnetic anisotropy depending on a doping level has never been observed in any other magnetic materials thus far, highlighting one of the unique aspects of the current system. Figure 2C shows the corresponding evolution of the symmetrized magnetoresistance (MR), which is fully consistent with that of AHE: A butterfly-shaped MR and a square-shaped AHE were commonly observed both at phase I and phase III, while a negative, broad, and less hysteretic MR emerged at phase II, where AHE exhibited nonlinear behavior without hysteresis as mentioned above.

**Fig. 3.**
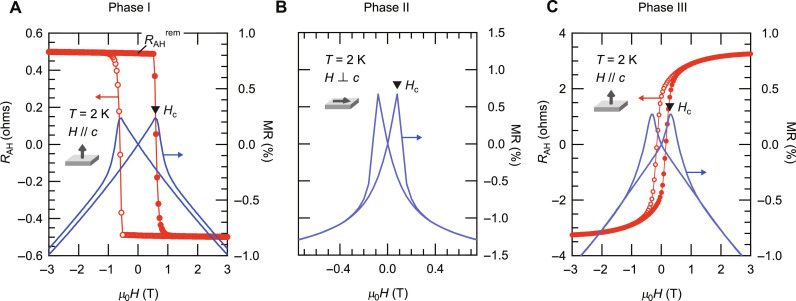
Evaluation of the magnetic anisotropy. (**A** to **C**) The magnetic-field dependences of *R*_AH_ (red) and MR (blue) taken at *T* = 2 K at (A) phase I (device B, *V*_G_ = 0 V), (B) phase II (device C, *V*_G_ = 3.4 V), and (C) phase III (device B, *V*_G_ = 3.4 V). The magnetic fields were aligned to be parallel to the easy axis directions. *R*_AH_ stands for the AH resistance, which is calculated from *R_yx_* by subtraction of the OH component. Note that phase II does not have the *R*_AH_ data, which was not measured because no AHE signal is expected for the in-plane fields. *R*_AH_^rem^ corresponds to *R*_AH_ at zero field [see (A)]. *H*_c_ corresponds to the coercive field.

The obtained experimental results support the idea that the magnetic anisotropy was switched two times across the three phases. In particular, a square-shaped AHE and a butterfly-shaped MR with clear hysteresis at phase I and phase III provide decisive evidence that those two phases host the out-of-plane magnetic anisotropy. Figure 3 (A and C) summarizes the AHE (red) and MR (blue) signals at phase I and phase III, respectively, showing a good correspondence with each other. The peak magnetic field of the MR curve should correspond to the coercive field (*H*_c_), at which the magnetization changes its direction from one to the other along the external field direction. On the other hand, a signature of the in-plane anisotropy at phase II characterized with a nonlinear AHE and a broad MR with negligible hysteresis shown in Fig. 2 (B and C) might be rather elusive. To address this issue, we performed additional MR measurements at phase II with the in plane magnetic fields. Figure 3B shows the corresponding MR data taken at *T* = 2 K with the in-plane fields at phase II. It turned out that the shape of MR transformed from a broad, less hysteretic one with the out-of-plane fields (Fig. 2C) to a butterfly-shaped, hysteretic one with the in-plane fields (Fig. 3B), decisively elucidating that phase II has the in-plane magnetic anisotropy.

**Fig. 4.**
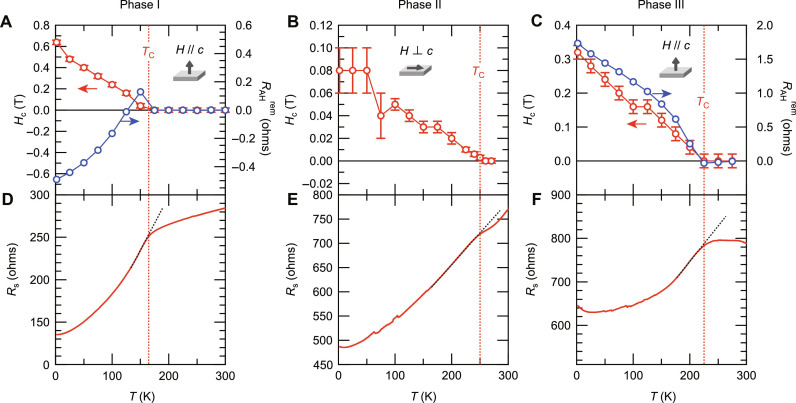
Determination of *T*_C_. (**A** to **C**) The temperature dependences of *H*_c_ (red) and *R*_AH_^rem^ (blue) at (A) phase I (device B, *V*_G_ = 0 V), (B) phase II (device C, *V*_G_ = 3.4 V), and (C) phase III (device B, *V*_G_ = 3.4 V). The magnetic fields were aligned to be parallel to the easy axis directions, the same settings as in Fig. 3. All the MR curves used to determine *H*_c_ are shown in Supplementary Text. The AHE data used to deduce *R*_AH_^rem^ are shown in Supplementary Text. (**D** to **F**) The corresponding *R*_s_*-T* curves taken at (D) phase I (device B, *V*_G_ = 0 V), (E) phase II (device C, *V*_G_ = 3.4 V), and (F) phase III (device B, *V*_G_ = 3.4 V). The red dotted lines represent the kink temperatures defined in the *R*_s_-*T* curves, exactly matching to the onset temperatures of *H*_c_ and *R*_AH_^rem^.

The red symbols in Fig. 4 (A to C) show the temperature dependences of *H*_c_ at phase I, phase II, and phase III, respectively, determined by the MR curves taken at each temperature (see Supplementary Text). Note that the magnetic fields were set to be parallel to the easy axis directions (the same settings as in Fig. 3). Also shown by the blue symbols in Fig. 4 (A and C) are those of *R*_AH_^rem^ at phase I and phase III, respectively, which is defined as the AH resistance (*R*_AH_) value at zero field (see Fig. 3A) and deduced from the AHE data taken at each temperature (see Supplementary Text). The *H*_c_ and *R*_AH_^rem^ were verified to have the common onset temperature, corresponding to the onset of the spontaneous magnetization, which is nothing but *T*_C_. On the basis of those magneto-transport results, we conclude that *T*_C_ evolved from the lowest level at phase I to the highest level at phase II and then to the intermediate level at phase III with increasing *V*_G_, demonstrating non-monotonous variation against a doping level. Owing to a good correspondence between *T*_C_ determined by the magneto-transport measurements and the kink temperature in the *R*_s_-*T* curve as shown in Fig. 4 (D to F), we could define *T*_C_ at each *V*_G_ from the *R*_s_-*T* data shown in Fig. 2A and discuss the evolution of *T*_C_ against a doping level more in detail.

### Magnetic phase diagram of Cr_3_Te_4_

**Fig. 5.**
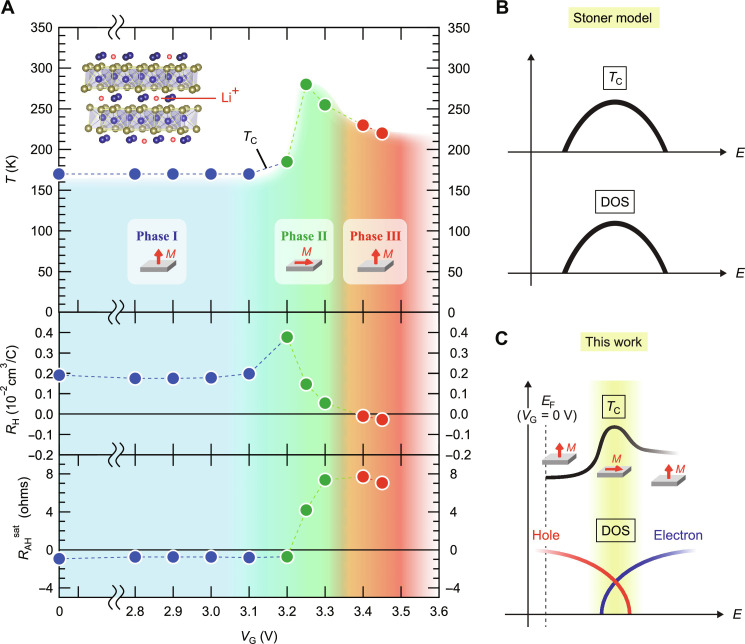
Magnetic phase diagram of Cr_3_Te_4_. (**A**) The evolutions of *T*_C_, the Hall coefficient (*R*_H_), and *R*_AH_ at the saturated regime (*R*_AH_^sat^) as a function of *V*_G_. The blue, green, and red regions are corresponding to the three distinct phases: phase I, phase II, and phase III, respectively. All the values plotted in this phase diagram were obtained from the data on device A shown in Fig. 2. The inset shows a schematic of Li-intercalated Cr_3_Te_4_. (**B** and **C**) Schematic illustrations of the correspondence between *T*_C_ and the density of states (DOS) for (B) a simple Stoner model and (C) this work.

Figure 5A summarizes the magnetic phase diagram of Cr_3_Te_4_ uncovered by the present ion-gating experiments, where *T*_C_, *R*_H_, and *R*_AH_ at the saturated regime (*R*_AH_^sat^) are plotted as a function of *V*_G_, highlighting the unique feature of this material system. Phase I is the initial state of the MBE-grown Cr_3_Te_4_, which is characterized with *T*_C_ of ~170 K and large out-of-plane magnetic anisotropy. With increasing a doping level, the system enters phase II, where *T*_C_ is largely enhanced near to room temperature. This phase change accompanies the switching of the magnetic anisotropy from the out-of-plane type to the in-plane one as schematically illustrated in the inset. On the other hand, *R*_H_ increases as the depletion of hole carrier proceeds and suddenly drops, showing a peak-like structure within phase II, which accompanies the sign change of *R*_AH_^sat^. *R*_H_ lastly reaches to a negative value in phase III, where *T*_C_ is slightly decreased and the anisotropy returns back to the out-of-plane one. The large enhancement of *T*_C_, the emergence of the in-plane magnetic anisotropy, the singular behavior in *R*_H_, and the sign change of *R*_AH_^sat^ occur synchronously within phase II, suggesting the same origin. Considering that *R*_AH_^sat^ changes its sign exactly when *R*_H_ starts to decrease, a peak-like structure in *R*_H_ is reflecting a semimetallic nature of this material system, and phase II corresponds to the specific regime where *E*_F_ is located nearly at the band crossing points. Such a non-monotonous variation in *T*_C_ could not be explained by a simple Stoner model, where *T*_C_ should be proportional to the density of states at *E*_F_ as schematically drawn in Fig. 5B, which is totally different from our observations, where *T*_C_ shows maximum near the band crossing points as illustrated in Fig. 5C, accompanying the two-step switching of the magnetic anisotropy. A similar carrier-density dependence of *T*_C_ was observed in one of magnetic topological insulators, Mn-doped (Bi,Sb)_2_(Se,Te)_3_, where *T*_C_ shows the maximum value exactly when *E*_F_ is positioned at the Dirac point (*18*), implying that the observed anomalous carrier-density dependence of *T*_C_ might capture a general feature of magnetism in magnetic semimetals.

## DISCUSSION

Here, we discuss a possible origin of magnetism in the current system based on our observations. First, the strongly carrier-density–dependent nature indicates that the magnetism in Cr_3_Te_4_ should be governed by the itinerant carriers rather than the local exchange interaction. Second, a non-monotonous variation in *T*_C_ does not agree with a simple Stoner model as mentioned above, which is clearly distinct from the previous gating results on a vdW magnetic metal, Fe_3_GeTe_2_ (*22*). Moreover, this unusual behavior is also unexpected within the framework of the conventional carrier–mediated RKKY mechanism (*39*–*41*), which supports a positive correlation between the ferromagnetic interaction (and thus *T*_C_) and the carrier density at least in the low–carrier-density regime as widely demonstrated in diluted magnetic semiconductors (*19*–*21*). On the other hand, for magnetic semimetals hosting a band crossing point in the momentum space, the valence-electron–mediated Van Vleck mechanism should have a large contribution (*42*), where an energy gain associated with the generation of the magnetic susceptibility due to the band hybridization plays an essential role as often discussed in magnetic topological insulators (*43*, *44*). Given that the effect of the band mixing should be maximized when *E*_F_ is positioned exactly at the band crossing points, the magnetism associated with the Van Vleck mechanism should be highly sensitive to *E*_F_, and it should be weakened when *E*_F_ is moved far away from the crossing points. In addition, considering that the band hybridization naturally generates orbital angular momentum in the momentum space, the magnetic anisotropy could be also modulated near the crossing points, which is exactly what we observed in our experiments. Together, we consider that the large enhancement of *T*_C_ and the sudden change in the magnetic anisotropy at phase II should be associated with the activation of the Van Vleck mechanism near the band crossing points. Considering that *T*_C_ should be determined as the sum of the exchange interactions in the system, the large enhancement of *T*_C_ at phase II is somewhat within our expectation because the Van Vleck mechanism should purely “add” an extra contribution to another mechanism most likely the RKKY mechanism that governs the magnetism in Cr_3_Te_4_ in the entire regime even far away from the crossing points.

In summary, the present study demonstrates the successful modulation of the magnetic properties of a vdW magnetic semimetal Cr_3_Te_4_ by an ion-gating technique, where the fundamental properties of a ferromagnet, *T*_C_ and magnetic anisotropy, are largely tuned by *V*_G_. In addition, on the basis of the systematic and synchronous variations of the magnetic properties and the transport coefficients including OHE and AHE, we could get an insight into the origin of magnetism in this material system, where the nontrivial contributions of itinerant electrons near the crossing points to magnetism should play a crucial role. The suggested scenario could be generally applicable to other magnetic semimetals, providing a perspective on designing a next generation 2D magnet with high-enough *T*_C_ and large-enough magnetic anisotropy that are required for future nanoscale 2D spintronics applications.

## MATERIALS AND METHODS

### Thin film growth

Cr_3_Te_4_ epitaxial thin films were grown on sapphire (001) substrates by MBE (EIKO Engineering) by following our previously established growth process (*34*). The growth temperature was set to 450°C. During the growth, Cr was supplied by an electron-beam evaporator with the evaporation rate below 0.05 Å/s, while Te was supplied by a Knudsen cell with the rate of ~3 Å/s throughout the growth process. All the films were annealed at 450°C for 60 min after the growth with the same Te flux to improve the crystalline quality.

### Device fabrication

The ion-gating devices used in this study were fabricated by a standard process without lithography as follows. Ni (10 nm thick) and Au (100-nm thick) were deposited onto the films as source/drain electrodes and voltage probes by an electron-beam evaporator through a metal mask with a Hall bar pattern, followed by mechanical scratching between each electrode to isolate voltage probes and define a channel size to be 100 μm in length and 300 μm in width. Subsequently, a Pt plate was placed above a channel region as a gate electrode, and a droplet of a polymer electrolyte was inserted in between a channel and a Pt plate just before the measurements. A polymer electrolyte was made of polyethylene glycol (PEG) (*M*_w_ = 600, Wako) and LiClO_4_ (Sigma-Aldrich), which was heated at 80°C under vacuum before use. The ratio of Li to O in the PEG was set to 1:20.

### Transport measurements

The electrical transport properties of the ion-gating devices were characterized by combination of a source-measure unit (Agilent Technologies, B2912A) and voltmeters (Keithley Instruments, 2182A) at different temperatures and magnetic fields under different *V*_G_ in Physical Property Measurement System (Quantum Design, PPMS). *V*_G_ was applied at *T* = 330 K under high vacuum condition (<10^−4^ torr). After cooling the system down to *T* = 250 K, the sample chamber was purged with helium gas to improve the stability of temperature.

### Band structure calculation

We used Vienna Ab initio Simulation Package (*45*, *46*) for the electronic structure calculations of Cr_3_Te_4_, where the crystal structure reported in (*24*) was assumed. Here, the exchange-correlation functional proposed by Perdew *et al*. (*47*), and pseudopotentials with the projector augmented wave basis (*48*) were used. In the calculations with spin-orbit coupling, we assumed the out-of-plane magnetization. The 12 × 12 × 12 Monkhorst-Pack *k*-grid and 500 eV as a cutoff energy of the plane wave basis set were used in the self-consistent field calculations. On top of the electronic structure calculations, we calculated the AH conductivity based on the Wannier tight-binding model constructed through Wannier90 package (*49*, *50*). Here, we used 88 Bloch states evaluated on the 8 × 8 × 8 uniform *k*-grid in the disentanglement process. The trial orbitals were set to Cr-*d* and Te-*p* orbitals, and the total number of orbitals was 54 including spin degrees of freedom. In the AH conductivity calculations, the Berry curvatures were evaluated on the 50 × 50 × 50 course *k*-grid with five times finer adaptive *k*-mesh based on the adaptive mesh refinement method.

## Supplementary Materials

### This PDF file includes:

Supplementary Text Figs. S1 to S8 References
