# Supplementary material for: Band-driven switching of magnetism in a van der Waals magnetic semimetal
Source: Sci Adv. 2024 Apr 12;10(15):eadk1415. doi: 10.1126/sciadv.adk1415 (PMC11014443; doi:10.1126/sciadv.adk1415)
Supplement: Supplementary file 1 — Supplementary Text Figs. S1 to S8 References [file sciadv.adk1415_sm.pdf]

Science Advances
AAAS

Supplementary Materials for

Band-driven switching of magnetism in a van der Waals magnetic semimetal

Hideki Matsuoka *et al.*

Corresponding author: Yoshihiro Iwasa, iwasa@ap.t.u-tokyo.ac.jp; Masaki Nakano, nakano@ap.t.u-tokyo.ac.jp

Sci. Adv. **10**, eadk1415 (2024)
DOI: 10.1126/sciadv.adk1415

This PDF file includes:

Supplementary Text
Figs. S1 to S8
References

Supplementary Text

A. Thin film characterizations.

The crystallinities of the obtained films were characterized by a reflection high-energy electron diffraction system (EIKO Engineering) and by a four-circle X-ray diffractometer (PANalytical, Empyrean). Figure S1 shows the out-of-plane x-ray diffraction patterns of a typical as-grown sample used in this study. The strong diffraction peaks were observed at around 14.6° with clear Laue oscillation, indicating high crystalline coherence along the out-of-plane direction. The thickness (layer number) of the obtained film was calculated from the Laue oscillation periods, which was defined as the number of the $CrTe_2$ layer. Magnetization measurements were performed by Magnetic Property Measurement System (Quantum Design, MPMS). We note that the structural phase of our samples was identified as Cr_3Te_4 by structural, magnetic, and transport characterizations in the previous study (*34*).

B. Reversibility of the gating effects on Cr_3Te_4.

We confirmed that the observed gating effects were highly reversible within our experimental condition. Figure S2A shows the R_s-T curves of Device E before and after the gating experiments, where $V_G = 0$ V and $V_G = 3.7$ V correspond to Phase I and Phase III, respectively, for this particular device. By setting V_G back to 0 V after applying V_G = 3.7 V, both the R_s-T curve (Fig. S2A) and the R_s value at $T = 330$ K (Fig. S2B) almost returned back to those at $V_G = 0$ V before gating. Considering that the destructive chemical reactions such as Te removal and/or chemical etching should accompany an irreversible device operation, the observed reasonably-good reversibility suggests that electron doping was induced by a reversible Li intercalation process.

C. Reproducibility of the gating effects on Cr_3Te_4.

We also confirmed that the observed gating effects were highly reproducible among different devices. In this study, we examined five devices in total. Figures S3A-D summarize the gating effects on (A) Device A, (B) Device B, (C) Device C, and (D) Device D, respectively, where the evolutions of the AHE signals against a doping level at the lowest temperature ($T = 2$ K) are displayed. All the devices exhibited the gate-induced phase transformation from Phase I to Phase II, demonstrating very high reproducibility. Note that the exact V_G value at each phase was slightly different among different devices, which should be originating from the fact that the effective voltage applied to the samples could be different depending on the local geometry of the device even though the same V_G was applied to the samples. Each device was prepared and examined for a specific

purpose. We here summarize the purpose of the experiments for each device and the correspondence between the device and the displayed items.

Device A: examination of the overall gating effects
Layer number of Cr_3Te_4: 25 L
Data: Figs. 2A, 2B, 2C, 5A, and S3A.

Device B: determination of T_C and magnetic anisotropy at Phase I and Phase III
Layer number of Cr_3Te_4: 25 L
Data: Figs. 3A, 3C, 4A, 4C, 4D, 4F, S3B, S4A, S4C, S4D, S4F, S4G, S4I, S5A, S5C, S6B, and S6D.

Device C: determination of T_C and magnetic anisotropy at Phase II
Layer number of Cr_3Te_4: 25 L
Data: Figs. 3B, 4B, 4E, S3C, S4B, S4E, and S4H.

Device D: examination of AHE at Phase II
Layer number of Cr_3Te_4: 24 L
Data: Figs. S3D, S5B, S6A, and S6C.

Device E: examination of the reversibility
Layer number of Cr_3Te_4: 15 L
Data: Figs. S2A and S2B.

D. The detailed magnetoresistance data.

In order to determine T_C, we performed the MR measurements at various temperatures with the magnetic fields applied parallel to the easy axis directions. Figures S4A-C show the MR curves at Phase I, Phase II, and Phase III, respectively, which are the same as those shown in Figs. 3A-C in the main text. We here define ΔMR as the difference in the MR signals taken with the opposite field-sweep directions (Figs. S4D-F), whose peak magnetic fields should correspond to H_c. Considering that the non-zero ΔMR signal should be relevant to the existence of the spontaneous magnetization, we should be able to determine T_C by tracking the temperature dependence of ΔMR. Figures S4G-I correspond to the ΔMR curves measured at different temperatures, which decreased monotonously with increasing the temperature and disappeared at T_C. The red data shown in Figs. 4A-C in the main text were deduced from Figs. S4G-I.

E. The detailed anomalous Hall effect data.

Figures S5A-C show the temperature dependences of AHE at each phase. The data at T = 2 K for Phase I and Phase III shown in Figs. S5A and S5C are the same as those shown in Figs. 3A and 3C in the main text. The temperature dependence of AHE at Phase I is essentially the same as those reported in our previous study (*34*), including the sign change near T = 100 K. In contrast, the AHE signals at Phase II and Phase III turned out to exhibit rather monotonous temperature dependences without sign reversal. The blue data shown in Figs. 4A and 4C in the main text were deduced from Figs. S5A and S5C.

F. The Arrott plot analysis.

The Arrott plot analysis is commonly employed to determine T_C from the AHE data. Here, we present the results of the Arrott plot analysis on the AHE data obtained in this study. However, we note that this analysis should be originally applied to the magnetization data, and therefore, when applied to the AHE data, we should consider the contribution of OHE. In conventional ferromagnetic metals, the contribution of OHE is negligible due to its high carrier density, and therefore, the Arrott plot analysis should provide rather reliable T_C. However, in magnetic semimetals, the contribution of OHE is usually large and cannot be ignored. To increase the accuracy of the analysis, we applied this analysis to the AHE data after the subtraction of the OHE components.

Figures S6A and S6B show the Arrott plot for Phase II and Phase III, respectively. The contribution of OHE, R_n, was assumed to be a linear component against the magnetic field at the lowest temperature (T = 2 K), and subtracted from the measured R_{yx} for the analysis. For all the obtained curves, linear fits were applied to obtain the intercepts of the extrapolations to the vertical axis, whose sign-reversal-temperatures provide T_C. Figures S6C and S6D show T_C obtained from the Arrott plot analysis, T_C (Arrott plot), together with those deduced from the R-T curves, T_C (R-T), and/or the magneto-transport (MR and AHE) measurements, T_C (MR) and T_C (AHE), for Phase II and Phase III, respectively, showing good correspondences. For Phase III, T_C defined by the Arrott plot was higher than those defined by other methods, but such a deviation is often observed in 2D magnetic materials, presumably associated with the formation of the magnetic domains at zero magnetic field (*22*, *52*).

G. Calculations of the band structure and anomalous Hall conductivity.

We confirmed that Cr_3Te_4 hosts a semimetallic band region near E_F by the first-principles calculations based on the density functional theory (DFT). Figures S7A and S7B show the results of the DFT calculations at k_z = 0.0 and k_z = 0.5, respectively, showing a

semimetallic band region near the M point as highlighted by the yellow hatched area. This clearly proves that Cr_3Te_4 is one of the magnetic semimetals hosting both electron- and hole-like bands that are crossing near E_F. Figure S7C shows the magnified views of the band structures near the M point for different k_z values, which is similar to those shown in the previous study (*35*). Given that the band calculations in the previous study were performed on $CrTe_2$, which is a common backbone of $Cr_{1+\delta}Te_2$, our calculation results provide us an important implication that the host $CrTe_2$ layer would govern a semimetallic nature of this material system, where the band crossing around the M point plays a key role. On the other hand, the electronic structure around the Γ point is mainly represented by the multiple hole bands near E_F, which is also consistent with the previous study (*35*). Importantly, as revealed by the ARPES measurements in the previous study, E_F of the as-grown $Cr_{1+\delta}Te_2$ thin film is lower than the calculated E_F, possibly due to the hole doping by the excess Te (*34*).

Figure S8 shows the anomalous Hall conductivity, σ_{AH}, as a function of energy, E-E_F, calculated from the band structure of Cr_3Te_4. σ_{AH} exhibits a sign change at around E-E_F = -0.5, and shows positive values near E-E_F = 0. Considering that the doping level of our as-grown film is through to be lower than that of bulk Cr_3Te_4 (*34*), E_F of the pristine ungated state should be located a bit below E-E_F = 0. Consequently, the sign change of the AHE signal from negative to positive upon electron doping that we observed in our experiments would qualitatively agree with the calculation results.

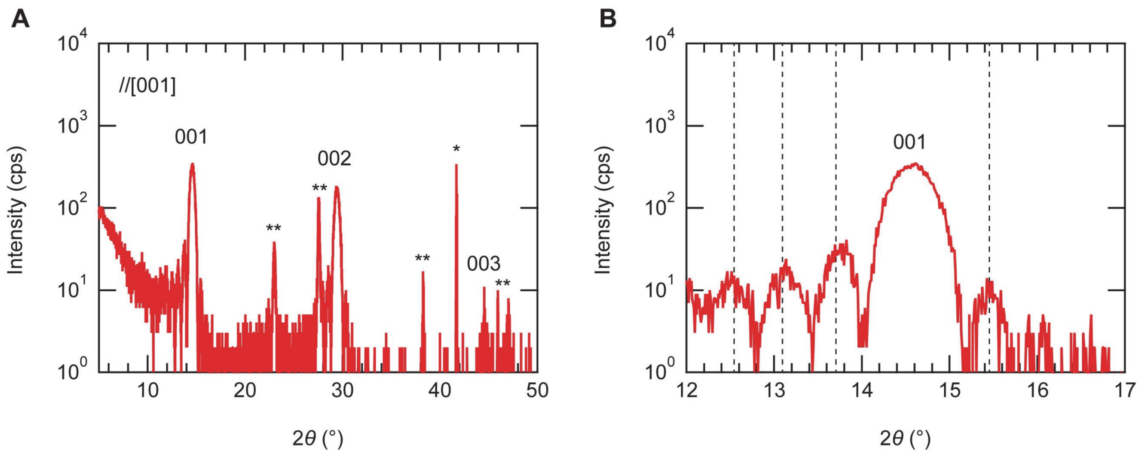

Fig. S1. The out-of-plane XRD pattern of the as-grown Cr_3Te_4 epitaxial film. (**A** and **B**) The out-of-plane XRD pattern of the as-grown sample used for Device B and Device C. The single and double asterisks are for the diffractions from the substrate and the Te capping layer, respectively. The thickness (layer number) of this sample was determined to be 25 L from the Laue oscillation periods.

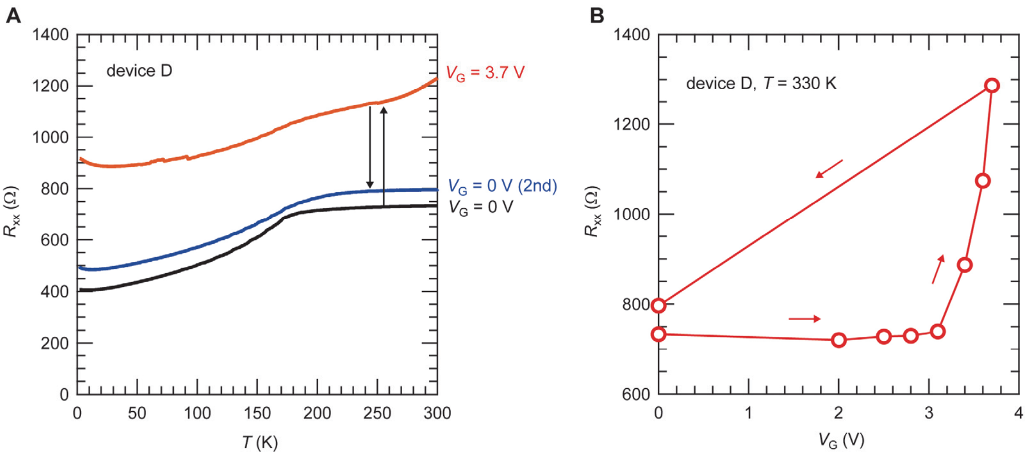

Fig. S2. Reversibility of the gating effects on Cr_3Te_4. (**A**) The R_s-T curves of Device E taken before gating ($V_G = 0$ V), under gating ($V_G = 3.7$ V), and after gating [$V_G = 0$ V (2nd)]. (**B**) The V_G-dependence of R_s at $T = 330$ K.

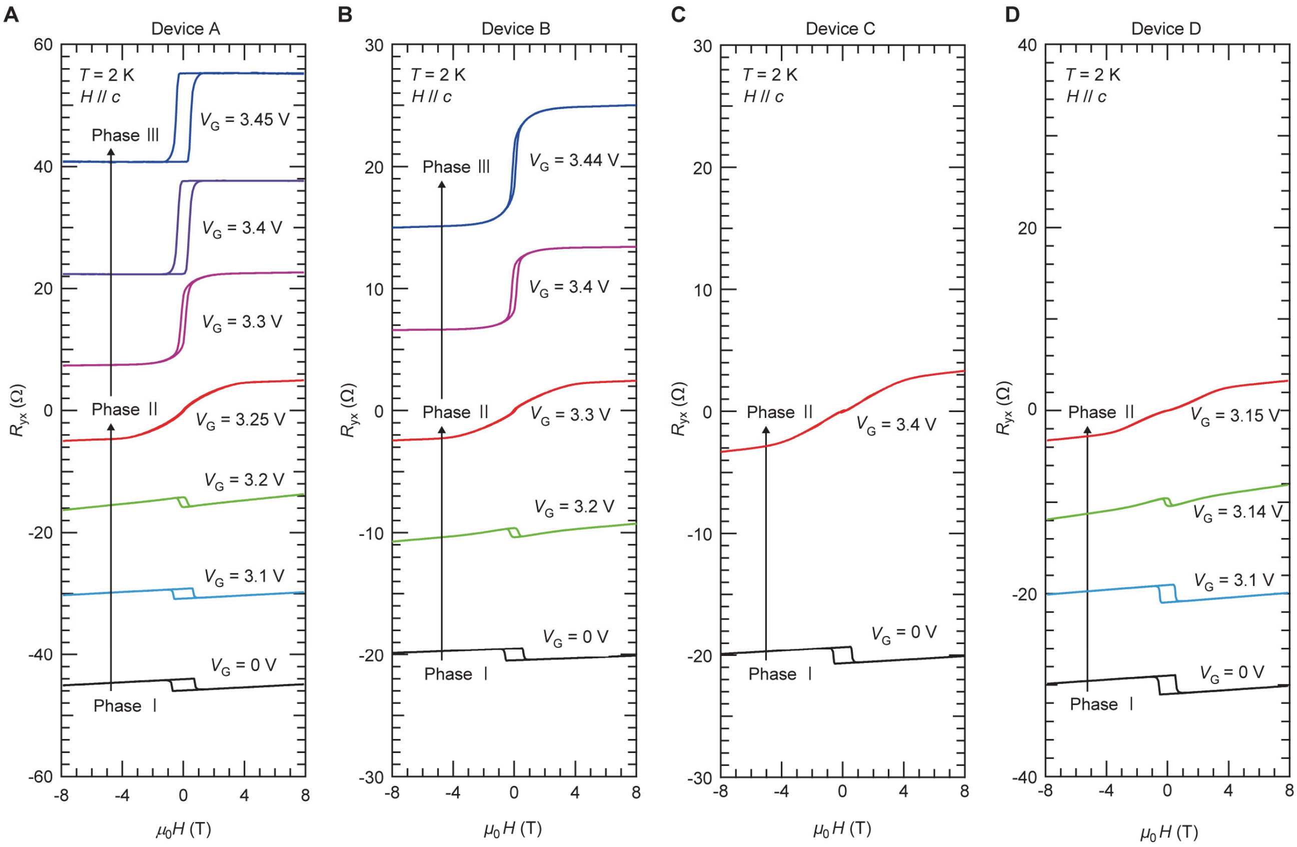

Fig. S3. Reproducibility of the gating effects on Cr_3Te_4. (**A** to **D**) The AHE data of (A) Device A, (B) Device B, (C) Device C, and (D) Device D, respectively, taken at $T = 2$ K at each V_G. The magnetic fields were set to be the out-of-plane directions. All the data are vertically shifted for clarity.

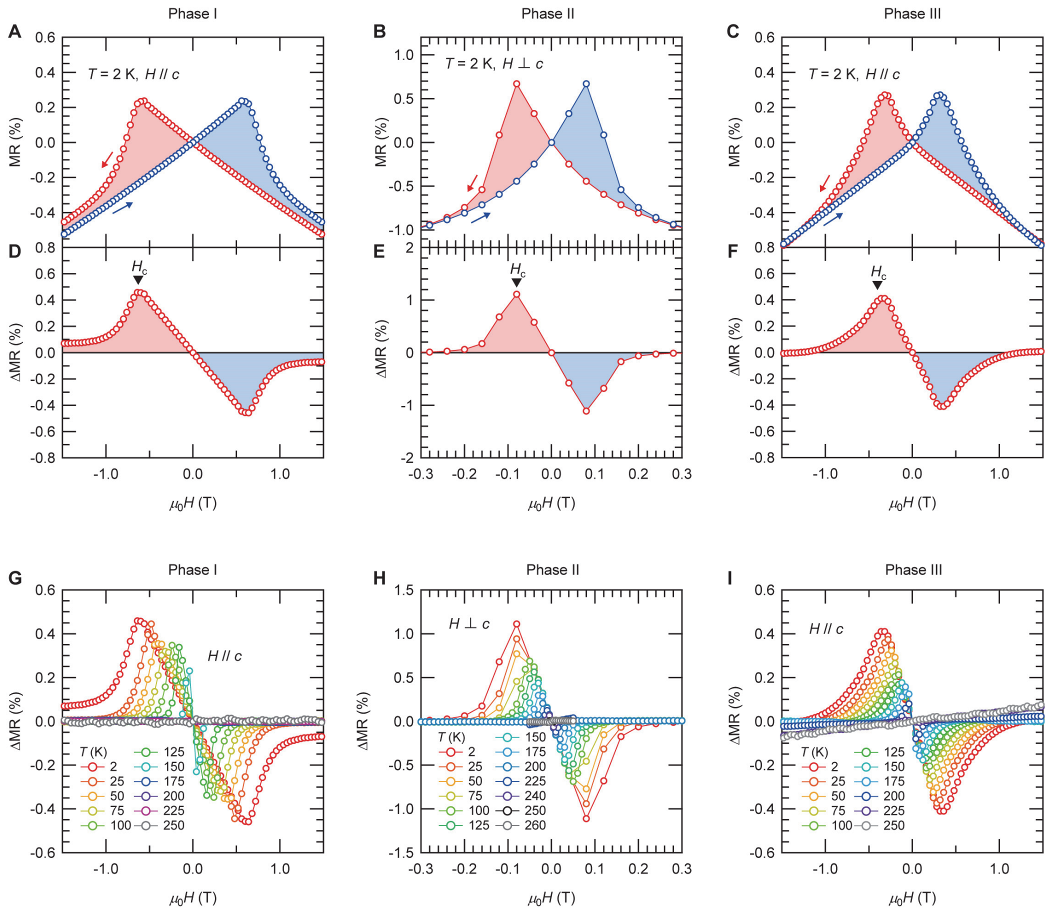

Fig. S4. The detailed MR data at each phase. (**A** to **C**) The symmetrized MR curves at $T = 2$ K for (A) Phase I (Device B, $V_G = 0$ V), (B) Phase II (Device C, $V_G = 3.4$ V), and (C) Phase III (Device B, $V_G = 3.4$ V). The magnetic fields were aligned to be parallel to the easy axis directions (H // c for Phase I and Phase III, and $H \perp c$ for Phase II). (**D** to **F**) The difference in the MR signals at $T = 2$ K taken with the opposite field-sweep directions (ΔMR) for (D) Phase I, (E) Phase II, and (F) Phase III. The inverse triangles indicate H_c, where ΔMR shows the maximum value. (**G** to **I**) The temperature dependence of ΔMR for (G) Phase I, (H) Phase II, and (I) Phase III. The H_c data shown in Figs. 4A-C in the main text were derived from Figs. S4G-I.

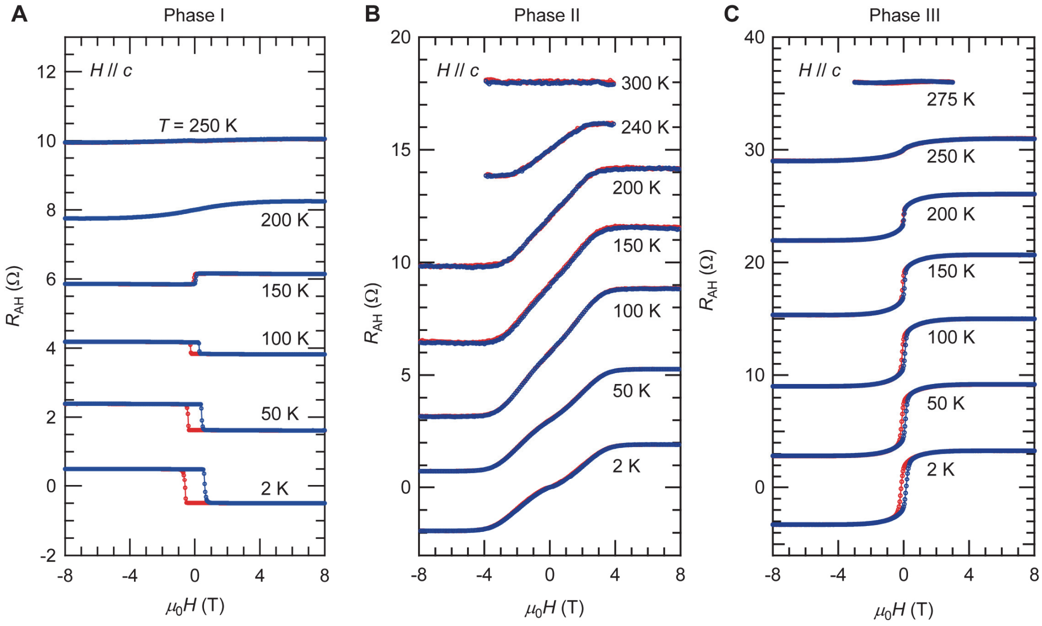

Fig. S5. The detailed AHE data at each phase. (**A** to **C**) The anti-symmetrized R_{AH} data at different temperatures for (**A**) Phase I (Device B, V_G = 0 V), (**B**) Phase II (Device D, V_G = 3.15 V), and (**C**) Phase III (Device B, V_G = 3.4 V). The magnetic fields were set to be the out-of-plane directions. The R_{AH}^{rem} data shown in Figs. 4A and 4C in the main text were derived from Figs. S5A and S5C.

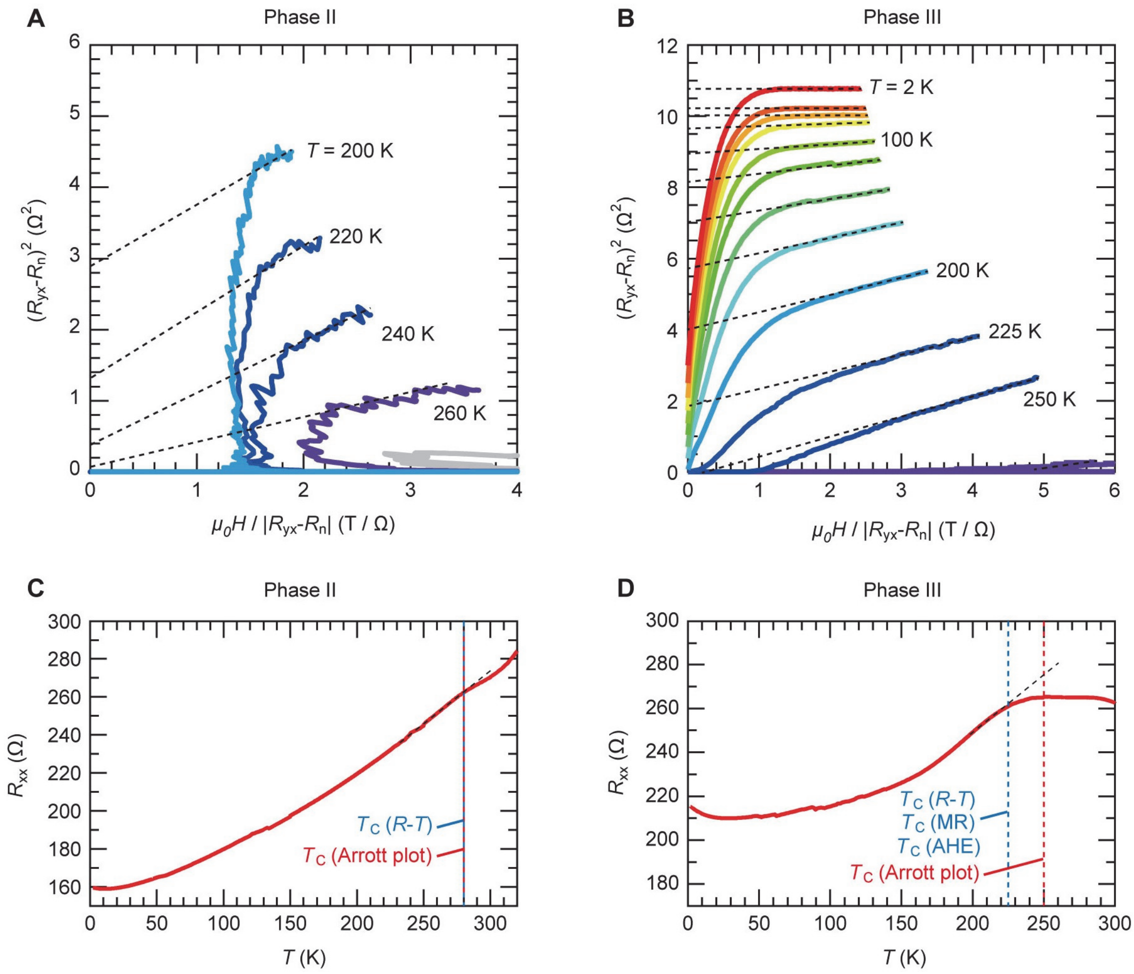

Fig. S6. Determination of T_C by the Arrott plot analysis. (**A** and **B**) The Arrott plots of the AHE data at (A) Phase II (Device D, V_G = 3.15 V) and (B) Phase III (Device B, V_G = 3.4 V). The OHE signals obtained by the linear fittings at T = 2 K were subtracted to improve the reliability of the analysis. (**C** and **D**) The R-T curves at (C) Phase II (Device D, V_G = 3.15 V) and (D) Phase III (Device B, V_G = 3.4 V). The dashed lines correspond to T_C obtained either from the Arrott plot analysis (red) or from other methods including the R-T, MR, and AHE measurements (blue).

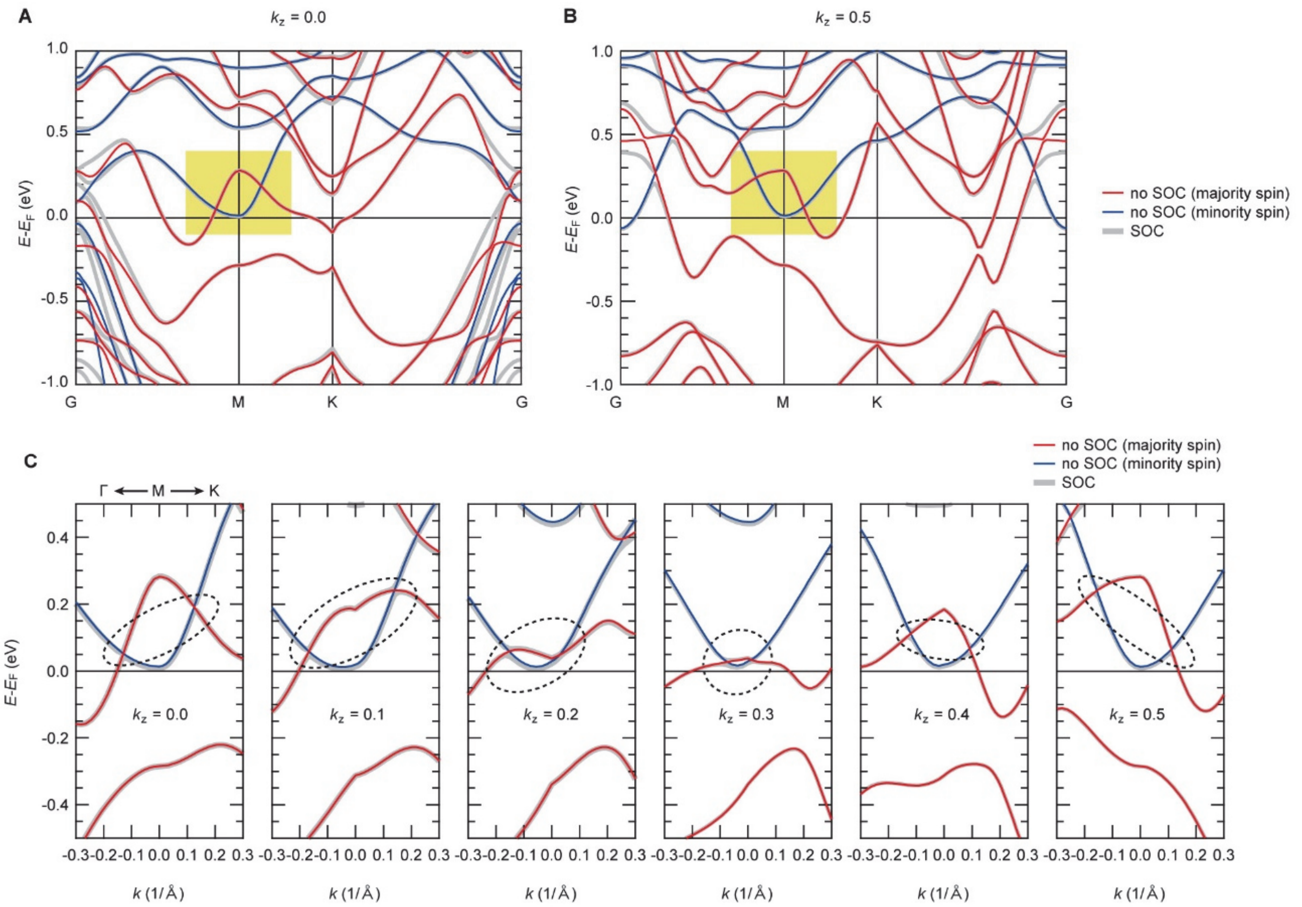

Fig. S7. The band structure of Cr_3Te_4. (**A** and **B**) The band structure of Cr_3Te_4 at (A) k_z = 0.0 and (B) k_z = 0.5. The red and blue lines correspond to the band dispersions for the majority and minority spins, respectively, without spin-orbit coupling (SOC), while the gray line corresponds to the band dispersion with SOC. The yellow hatched areas indicate a semimetallic band region near the M point. To establish a correspondence with the band structure of $Cr_{1+\delta}Te_2$ shown in the previous study (*35*), we employed the notations that are often used for a hexagonal Brillouin zone corresponding to the unit cell of the $CrTe_2$ layer. (**C**) The magnified views of the band structures near the M point at different k_z values. The black dashed lines highlighted the semimetallic band regions.

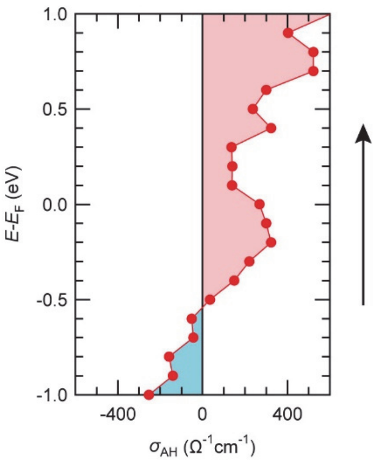

Fig. S8. The anomalous Hall conductivity of Cr_3Te_4. The anomalous Hall conductivity, σ_{AH}, as a function of energy, E-E_F, calculated from the band structure of Cr_3Te_4.

REFERENCES

1. M. Z. Hasan, C. L. Kane, Colloquium: Topological insulators. *Rev. Mod. Phys.* **82**, 3045–3067 (2010).

2. X.-L. Qi, S.-C. Zhang, Topological insulators and superconductors. *Rev. Mod. Phys.* **83**, 1057–1110 (2011).

3. C.-Z. Chang, J. Zhang, X. Feng, J. Shen, Z. Zhang, M. Guo, K. Li, Y. Ou, P. Wei, L.-L. Wang, Z.-Q. Ji, Y. Feng, S. Ji, X. Chen, J. Jia, X. Dai, Z. Fang, S.-C. Zhang, K. He, Y. Wang, L. Lu, X.-C. Ma, Q.-K. Xue, Experimental observation of the quantum anomalous Hall effect in a magnetic topological insulator. *Science* **340**, 167–170 (2013).

4. Y. Tokura, K. Yasuda, A. Tsukazaki, Magnetic topological insulators. *Nat. Rev. Phys.* **1**, 126–143 (2019).

5. X. Wan, A. M. Turner, A. Vishwanath, S. Y. Savrasov, Topological semimetal and Fermi-arc surface states in the electronic structure of pyrochlore iridates. *Phys. Rev. B* **83**, 205101 (2011).

6. G. Xu, H. Weng, Z. Wang, X. Dai, Z. Fang, Chern semimetal and the quantized anomalous Hall effect in HgCr2Se4. *Phys. Rev. Lett.* **107**, 186806 (2011).

7. E. Liu, Y. Sun, N. Kumar, L. Muechler, A. Sun, L. Jiao, S.-Y. Yang, D. Liu, A. Liang, Q. Xu, J. Kroder, V. Süß, H. Borrmann, C. Shekhar, Z. Wang, C. Xi, W. Wang, W. Schnelle, S. Wirth, Y. Chen, S. T. B. Goennenwein, C. Felser, Giant anomalous Hall effect in a ferromagnetic kagome-lattice semimetal. *Nat. Phys.* **14**, 1125–1131 (2018).

8. S. Nakatsuji, N. Kiyohara, T. Higo, Large anomalous Hall effect in a non-collinear antiferromagnet at room temperature. *Nature* **527**, 212–215 (2015).

9. L. Ye, M. Kang, J. Liu, F. von Cube, C. R. Wicker, T. Suzuki, C. Jozwiak, A. Bostwick, E. Rotenberg, D. C. Bell, L. Fu, R. Comin, J. G. Checkelsky, Massive Dirac fermions in a ferromagnetic kagome metal. *Nature* **555**, 638–642 (2018).

10. X. Lu, P. Stepanov, W. Yang, M. Xie, M. A. Aamir, I. Das, C. Urgell, K. Watanabe, T. Taniguchi, G. Zhang, A. Bachtold, A. H. MacDonald, D. K. Efetov, Superconductors, orbital magnets and correlated states in magic-angle bilayer graphene. *Nature* **574**, 653–657 (2019).

11. A. L. Sharpe, E. J. Fox, A. W. Barnard, J. Finney, K. Watanabe, T. Taniguchi, M. A. Kastner, D. Goldhaber-Gordon, Emergent ferromagnetism near three-quarters filling in twisted bilayer graphene. *Science* **365**, 605–608 (2019).

12. M. Serlin, C. L. Tschirhart, H. Polshyn, Y. Zhang, J. Zhu, K. Watanabe, T. Taniguchi, L. Balents, A. F. Young, Intrinsic quantized anomalous Hall effect in a moiré heterostructure. *Science* **367**, 900–903 (2020).

13. Y. Deng, Y. Yu, M. Z. Shi, Z. Guo, Z. Xu, J. Wang, X. H. Chen, Y. Zhang, Quantum anomalous Hall effect in intrinsic magnetic topological insulator $MnBi_2Te_4$. *Science* **367**, 895–900 (2020).

14. F. D. Haldane, Model for a quantum Hall effect without Landau levels: Condensed-matter realization of the “parity anomaly.”. *Phys. Rev. Lett.* **61**, 2015–2018 (1988).

15. K. Ohgushi, S. Murakami, N. Nagaosa, Spin anisotropy and quantum Hall effect in the *kagomé* lattice: Chiral spin state based on a ferromagnet. *Phys. Rev. B* **62**, R6065–R6068 (2000).

16. Z. Fang, N. Nagaosa, K. S. Takahashi, A. Asamitsu, R. Mathieu, T. Ogasawara, H. Yamada, M. Kawasaki, Y. Tokura, K. Terakura, The anomalous Hall effect and magnetic monopoles in momentum space. *Science* **302**, 92–95 (2003).

17. D. Xiao, M.-C. Chang, Q. Niu, Berry phase effects on electronic properties. *Rev. Mod. Phys.* **82**, 1959–2007 (2010).

18. J. G. Checkelsky, J. Ye, Y. Onose, Y. Iwasa, Y. Tokura, Dirac-fermion-mediated ferromagnetism in a topological insulator. *Nat. Phys.* **8**, 729–733 (2012).

19. H. Ohno, D. Chiba, F. Matsukura, T. Omiya, E. Abe, T. Dietl, Y. Ohno, K. Ohtani, Electric-field control of ferromagnetism. *Nature* **408**, 944–946 (2000).

20. Y. Yamada, K. Ueno, T. Fukumura, H. T. Yuan, H. Shimotani, Y. Iwasa, L. Gu, S. Tsukimoto, Y. Ikuhara, M. Kawasaki, Electrically induced ferromagnetism at room temperature in cobalt-doped titanium dioxide. *Science* **332**, 1065–1067 (2011).

21. F. Matsukura, Y. Tokura, H. Ohno, Control of magnetism by electric fields. *Nat. Nanotechnol.* **10**, 209–220 (2015).

22. Y. Deng, Y. Yu, Y. Song, J. Zhang, N. Z. Wang, Z. Sun, Y. Yi, Y. Z. Wu, S. Wu, J. Zhu, J. Wang, X. H. Chen, Y. Zhang, Gate-tunable room-temperature ferromagnetism in two-dimensional Fe_3GeTe_2. *Nature* **563**, 94–99 (2018).

23. I. A. Verzhbitskiy, H. Kurebayashi, H. Cheng, J. Zhou, S. Khan, Y. P. Feng, G. Eda, Controlling the magnetic anisotropy in $Cr_2Ge_2Te_6$ by electrostatic gating. *Nat. Electron.* **3**, 460–465 (2020).

24. H. Ipser, K. L. Komarek, K. O. Klepp, Transition metal-chalcogen systems viii: The Cr-Te phase diagram. *J. Less-Common Met.* **92**, 265–282 (1983).

25. F. K. Lotgering, E. W. Gorter, Solid solutions between ferromagnetic and antiferromagnetic compounds with NiAs structure. *J. Phys. Chem. Solid* **3**, 238–249 (1957).

26. M. Yamaguchi, T. Hashimoto, Magnetic properties of Cr_3Te_4 in ferromagnetic region. *J. Physical Soc. Japan* **32**, 635–638 (1972).

27. D. C. Freitas, R. Weht, A. Sulpice, G. Remenyi, P. Strobel, F. Gay, J. Marcus, M. Núñez-Regueiro, Ferromagnetism in layered metastable 1*T*-$CrTe_2$. *J. Phys. Condens. Matter* **27**, 176002 (2015).

28. X. Sun, W. Li, X. Wang, Q. Sui, T. Zhang, Z. Wang, L. Liu, D. Li, S. Feng, S. Zhong, H. Wang, V. Bouchiat, M. Nunez Regueiro, N. Rougemaille, J. Coraux, A. Purbawati, A. Hadj-Azzem, Z. Wang, B. Dong, X. Wu, T. Yang, G. Yu, B. Wang, Z. Han, X. Han, Z. Zhang,

Room temperature ferromagnetism in ultra-thin van der Waals crystals of 1T-$CrTe_2$. *Nano Res.* **13**, 3358–3363 (2020).

29. X. Zhang, Q. Lu, W. Liu, W. Niu, J. Sun, J. Cook, M. Vaninger, P. F. Miceli, D. J. Singh, S.-W. Lian, T.-R. Chang, X. He, J. Du, L. He, R. Zhang, G. Bian, Y. Xu, Room-temperature intrinsic ferromagnetism in epitaxial $CrTe_2$ ultrathin films. *Nat. Commun.* **12**, 2492 (2021).

30. R. Chua, J. Zhou, X. Yu, W. Yu, J. Gou, R. Zhu, L. Zhang, M. Liu, M. B. H. Breese, W. Chen, K. P. Loh, Y. P. Feng, M. Yang, Y. L. Huang, A. T. S. Wee, Room temperature ferromagnetism of monolayer chromium telluride with perpendicular magnetic anisotropy. *Adv. Mater.* **33**, e2103360 (2021).

31. H. Wu, W. Zhang, L. Yang, J. Wang, J. Li, L. Li, Y. Gao, L. Zhang, J. Du, H. Shu, H. Chang, Strong intrinsic room-temperature ferromagnetism in freestanding non-van der Waals ultrathin 2D crystals. *Nat. Commun.* **12**, 5688 (2021).

32. Y. Fujisawa, M. Pardo-Almanza, J. Garland, K. Yamagami, X. Zhu, X. Chen, K. Araki, T. Takeda, M. Kobayashi, Y. Takeda, C. H. Hsu, F. C. Chuang, R. Laskowski, K. H. Khoo, A. Soumyanarayanan, Y. Okada, Tailoring magnetism in self-intercalated $Cr_{1+\delta}Te_2$ epitaxial films. *Phys. Rev. Mater.* **4**, 114001 (2020).

33. K. Lasek, P. M. Coelho, P. Gargiani, M. Valvidares, K. Mohseni, H. L. Meyerheim, I. Kostanovskiy, K. Zberecki, M. Batzill, Van der Waals epitaxy growth of 2D ferromagnetic $Cr_{(1+\delta)}Te_2$ nanolayers with concentration-tunable magnetic anisotropy. *Appl. Phys. Rev.* **9**, 011409 (2022).

34. Y. Wang, S. Kajihara, H. Matsuoka, B. K. Saika, K. Yamagami, Y. Takeda, H. Wadati, K. Ishizaka, Y. Iwasa, M. Nakano, Layer-number-independent two-dimensional ferromagnetism in Cr_3Te_4. *Nano Lett.* **22**, 9964–9971 (2022).

35. Y. Fujisawa, M. Pardo-Almanza, C.-H. Hsu, A. Mohamed, K. Yamagami, A. Krishnadas, G. Chang, F.-C. Chuang, K. H. Khoo, J. Zang, A. Soumyanarayanan, Y. Okada, Widely tunable berry curvature in the magnetic semimetal $Cr_{1+\delta}Te_2$. *Adv. Mater.* **35**, e2207121 (2023).

36. Y. Yu, F. Yang, X. F. Lu, Y. J. Yan, Y.-H. Cho, L. Ma, X. Niu, S. Kim, Y.-W. Son, D. Feng, S. Li, S.-W. Cheong, X. H. Chen, Y. Zhang, Gate-tunable phase transitions in thin flakes of 1T-TaS_2. *Nat. Nanotechnol.* **10**, 270–276 (2015).

37. Y. Nakagawa, Y. Kasahara, T. Nomoto, R. Arita, T. Nojima, Y. Iwasa, Gate-controlled BCS-BEC crossover in a two-dimensional superconductor. *Science* **372**, 190–195 (2021).

38. M. Tang, J. Huang, F. Qin, K. Zhai, T. Ideue, Z. Li, F. Meng, A. Nie, L. Wu, X. Bi, C. Zhang, L. Zhou, P. Chen, C. Qiu, P. Tang, H. Zhang, X. Wan, L. Wang, Z. Liu, Y. Tian, Y. Iwasa, H. Yuan, Continuous manipulation of magnetic anisotropy in a van der Waals ferromagnet via electrical gating. *Nat. Electron.* **6**, 28–36 (2023).

39. M. A. Ruderman, C. Kittel, Indirect exchange coupling of nuclear magnetic moments by conduction electrons. *Phys. Rev.* **96**, 99–102 (1954).

40. T. Kasuya, A theory of metallic Ferro- and antiferromagnetism on Zener's model. *Progr. Theor. Phys.* **16**, 45–57 (1956).

41. K. Yoshida, Magnetic Properties of Cu-Mn Alloys. *Phys. Rev.* **106**, 893–898 (1957).

42. J. H. van Vleck, *The Theory of Electronic and Magnetic Susceptibilities* (Clarendon Press, 1932).

43. R. Yu, W. Zhang, H.-J. Zhang, S.-C. Zhang, X. Dai, Z. Fang, Quantized anomalous Hall effect in magnetic topological insulators. *Science* **329**, 61–64 (2010).

44. A. S. Núñez, J. Fernández-Rossier, Colossal anisotropy in diluted magnetic topological insulators. *Solid State Commun.* **152**, 403–406 (2012).

45. G. Kresse, J. Furthmüller, Efficiency of ab-initio total energy calculations for metals and semiconductors using a plane-wave basis set. *Comput. Mater. Sci.* **6,** 15–50 (1996).

46. G. Kresse, J. Furthmüller, Efficient iterative schemes forab initiototal-energy calculations using a plane-wave basis set, *Phys. Rev. B Condens. Matter* **54,** 11169–11186 (1996).

47. J. P. Perdew, K. Burke, M. Ernzerhof, Generalized gradient approximation made simple. *Phys. Rev. Lett.* **77**, 3865–3868 (1996).

48. G. Kresse, J. Furthmüller, From ultrasoft pseudopotentials to the projector augmented-wave method. *Phys. Rev. B* **59,** 1758–1775 (1999).

49. A. A. Mostofi, J. R. Yates, Y.-S. Lee, I. Souza, D. Vanderbilt, N. Marzari, wannier90: A tool for obtaining maximally-localised Wannier functions. *Comput. Phys. Commun.* **178**, 685–699 (2008).

50. G. Pizzi, V. Vitale, R. Arita, S. Blügel, F. Freimuth, G. Géranton, M. Gibertini, D. Gresch, C. Johnson, T. Koretsune, J. Ibañez-Azpiroz, H. Lee, J.-M. Lihm, D. Marchand, A. Marrazzo, Y. Mokrousov, J. I. Mustafa, Y. Nohara, Y. Nomura, L. Paulatto, S. Poncé, T. Ponweiser, J. Qiao, F. Thöle, S. S. Tsirkin, M. Wierzbowska, N. Marzari, D. Vanderbilt, I. Souza, A. A. Mostofi, J. R. Yates, Wannier90 as a community code: New features and applications. *J. Phys. Condens. Matter* **32**, 165902 (2020).

51. K. Momma, F. Izumi, *VESTA 3* for three-dimensional visualization of crystal, volumetric and morphology data. *J. Appl. Cryst.* **44**, 1272–1276 (2011)

52. Y. Deng, Z. Xiang, B. Lei, K. Zhu, H. Mu, W. Zhuo, X. Hua, M. Wang, Z. Wang, G. Wang, M. Tian, X. Chen, Layer-number-dependent magnetism and anomalous hall effect in van der Waals Ferromagnet Fe_5GeTe_2. *Nano Lett.* **22**, 9839–9846 (2022).
